# Hippo/YAP signaling pathway is involved in osteosarcoma chemoresistance

**DOI:** 10.1186/s40880-016-0109-z

**Published:** 2016-05-20

**Authors:** Dong-Yu Wang, Ya-Nan Wu, Jun-Qi Huang, Wei Wang, Meng Xu, Jin-Peng Jia, Gang Han, Bei-Bei Mao, Wen-Zhi Bi

**Affiliations:** Department of Orthopaedics and Rehabilitation, PLA General Hospital, Fuxing Rd 28, Beijing, 100853 P. R. China; State Key Laboratory of Brain and Cognitive Sciences, Institute of Biophysics, Chinese Academy of Sciences, Beijing, 100101 P. R. China

**Keywords:** Hippo, YAP, Methotrexate, Doxorubicin, Osteosarcoma, Chemoresistance

## Abstract

**Background:**

Osteosarcoma is the most common bone malignancy in children and adolescents, and 20%–30% of the patients suffer from poor prognosis because of individual chemoresistance. The Hippo/yes-associated protein (YAP) signaling pathway has been shown to play a role in tumor chemoresistance, but no previous report has focused on its involvement in osteosarcoma chemoresistance. This study aimed to investigate the role of the Hippo/YAP signaling pathway in osteosarcoma chemoresistance and to determine potential treatment targets.

**Methods:**

Using the Cell Titer-Glo Luminescent cell viability assay and flow cytometry analysis, we determined the proliferation and chemosensitivity of YAP-overexpressing and YAP-knockdown osteosarcoma cells. In addition, using western blotting and the real-time polymerase chain reaction technique, we investigated the alteration of the Hippo/YAP signaling pathway in osteosarcoma cells treated with chemotherapeutic agents.

**Results:**

Mammalian sterile 20-like kinase 1 (MST1) degradation was increased, and large tumor suppressor kinase 1/2 (LATS1/2) total protein levels were decreased by methotrexate and doxorubicin, which increased activation and nuclear translocation of YAP. Moreover, YAP increased the proliferation and chemoresistance of MG63 cells.

**Conclusions:**

The Hippo/YAP signaling pathway plays a role in osteosarcoma chemoresistance, and YAP is a potential target for reducing chemoresistance.

## Background

Osteosarcoma is the most common bone malignancy in children and adolescents. Since the introduction of neoadjuvant chemotherapy (chemotherapy before treatment) in the 1980s, the prognosis of osteosarcoma patients has improved markedly [1]. However, in the past 10 years, the survival rate has risen only slightly. Currently, the consensus is that poor chemotherapeutic effect on some patients is the primary obstacle to a higher survival rate of osteosarcoma patients [2]. Methotrexate and doxorubicin are the most commonly used drugs for the treatment of osteosarcoma, and resistance to them substantially decreases patients’ survival rates. Thus, many studies have investigated the mechanism of chemoresistance to methotrexate and doxorubicin, including impaired intracellular transportation components [3], inactivation of chemotherapeutic drugs [4], DNA self-repair enhancements [5], cell signaling transduction turbulence [6], microRNA dysregulation [7], and autophagy overreaction [8]. Nevertheless, for patients with osteosarcoma, the key mechanism of chemoresistance is still inconclusive. This motivated us to investigate alternative mechanisms for osteosarcoma chemoresistance.

The Hippo/yes-associated protein (YAP) signaling pathway was originally found in the *Drosophila* and has been proven to modulate organ size [9]. Its key components include mammalian sterile 20-like kinases 1/2 (MST1/2), salvador family WW domain-containing protein 1 (SAV1), large tumor suppressor kinases 1/2 (LATS1/2), YAP, transcriptional co-activator with PDZ-binding motif (TAZ), and transcriptional enhancer factor domain family members 1–4 (TEAD1–4) [10]. In humans, MST1/2 combines with SAV1 to form an activated complex that initiates LATS1/2 phosphorylation [11–13]. Once activated, LATS1/2 further promotes the signaling cascade by phosphorylating YAP at Ser127 or TAZ at Ser89. Phosphorylated YAP then binds to 14-3-3 protein and remains in the cytoplasm for degradation [14–16]. Dephosphorylated YAP translocates into the nucleus and binds to TEAD1–4, which activates downstream genes to support proliferation and inhibit apoptosis [17, 18]. The Hippo/YAP signaling pathway is involved in tumor chemoresistance. Mao et al. [19] reported that resistance to cisplatin is increased by YAP2 and silent mating type information regulation 2 homolog 1 (SIRT1) in hepatocellular carcinoma (HCC) cells, indicating that both YAP2 and SIRT1 protect HCC cells from the chemotherapeutic drug cisplatin. Similarly, ovarian cancer cells with knockdown of YAP/TEAD showed increased sensitivity to cisplatin, paclitaxel, and bleomycin [20]. Moreover, verteporfin, a YAP1 inhibitor, promotes sensitivity to 5-fluorouracil and docetaxel by directly inhibiting YAP1 and endothelial growth factor receptor in esophageal cancer cells [21]. Although many studies have investigated the role of the Hippo/YAP signaling pathway in chemoresistance, little is known about its function in osteosarcoma chemoresistance.

In this study, we try to find the role of Hippo/YAP signaling pathway in methotrexate- or doxorubicin-treated MG63 and U2OS osteosarcoma cells. We hope our experiments illustrate the function of YAP in osteosarcoma chemoresistance.

## Methods

### Cell cultures and reagents

Human osteosarcoma cell lines MG63 and U2OS were purchased from Cell Resource Center of Shanghai Institutes for Biological Sciences (Shanghai, China) and cultured in Minimal Essential Medium (Gibco, Waltham, Massachusetts, USA) with 10% fetal bovine serum (Biological Industries, Kibbutz Beit Haemek, Israel), 1% non-essential amino acid (Gibco), and penicillin/streptomycin (Gibco) in a humidified incubator under 95% air and 5% CO_2_ at 37 °C. All other cell culture materials were obtained from Gibco; all chemicals were obtained from Sigma-Aldrich (St. Louis, Missouri, USA).

### Virus packaging and infection

pQCXIH empty vector and pQCXIH-YAP constructs were gifts from Bin Zhao (Zhejiang University, China) [18]. pLKO empty vector and pLKO-YAP-knockdown expressing lentivirus were also constructed to obtain YAP knockdown cell lines. MG63 cells were infected with retrovirus that expresses empty vector and wild-type (WT) YAP separately to generate control and YAP-overexpressing stable cell lines. pLKO empty vector and pLKO-YAP-knockdown expressing lentivirus were used to treat MG63 cells to generate control and YAP-knockdown stable cell lines. Hygromycin and blasticidin screening was performed 48 h after infection.

### RNA extraction and quantitative real-time polymerase chain reaction (RT-PCR) analysis

Total RNA was isolated from cells using TRIzol reagent (Invitrogen-Life Technologies, Waltham, Massachusetts, USA). The reverse transcription products were used for RT-PCR with specific primers: MST1 (forward: 5′-AGACCTCCAGGAGATAATCAAAGA-3′; reverse: 5′-AGATACAGAACCAGCCCCACA-3′), Beta-Actin (forward: 5′-GTCTGCCTTGGTAGTGGATAATG-3′; reverse: 5′-TCGAGGACGCCCTATCATGG-3′).

### Immunofluorescence staining

MG63 and U2OS cells were fixed using 4% paraformaldehyde in phosphate buffered saline (PBS) for 15 min. After permeabilization, using 0.1% Triton X-100 in PBS and blocking in 3% bovine serum albumin in PBS, the cells were incubated in primary antibodies overnight at 4 °C. Alexa Fluor 546-conjugated secondary antibodies (Invitrogen-Life Technologies; 1:1000 dilution) were used. The samples were mounted using ProLong Gold Antifade Reagent with DAPI (Invitrogen-Life Technologies), and immunofluorescence was detected using an Olympus confocal microscope.

### Co-immunoprecipitation

Cells were collected, and proteins were solubilized in immunoprecipitation buffer (50 mM Tris pH 8.0, 150 mM NaCl, 1% NP40, 1% protease inhibitor cocktail) at 4 °C. Then, 1 mg of lysed protein was incubated with YAP antibody (ABclonal Biotech, A1002, College Park, Maryland, USA) and precipitated with protein A or G agarose (Upstate Biotechnology, Lake Placid, New York, USA) at 4 °C overnight. The immune complex was collected, washed three to five times, and probed with 14-3-3β antibody (Cell Signaling Technology, #9636, Danvers, Massachusetts, USA) and YAP antibody (ABclonal Biotech).

### Cell counting

MG63 cells were cultured in 96-well flat plates for 6 days. Before seeding, cell numbers were calculated using a countess automated cell counter (Invitrogen-Life Technologies) to keep the initial cell numbers equal. Culture media were rejuvenated every 48 h, and total cell numbers of cells were counted every 24 h. In this study, three independent experiments were performed.

### Cell viability assay

Cell Titer-Glo Luminescent Cell Viability Assay (Promega, Madison, Wisconsin, USA) was used to monitor cell total adenosine triphosphate (ATP). MG63 cells were seeded in a 96-well flat plate for 24 h and exposed to methotrexate (20 mM) or doxorubicin (10 μM) for another 24 h. Then, the Cell Titer-Glo reagent was added to the cells for 10 min. ATP was measured using a reporter luminometer. Relative cell viability was calculated according to the manufacturer’s instructions. This experiment was repeated three times.

### Cell apoptosis assay

Cell apoptosis was examined by flow cytometry analysis using the Annexin V-FITC and propidium iodide (PI) double-staining technique. MG63 cells were seeded in a 24-well culture plate at greater than 80% confluence and subjected to methotrexate (20 mM) or doxorubicin (10 μM) treatments for 24 h. Cells were stained following the Annexin V-fluorescein isothiocyanate (FITC) cell apoptosis detection kit’s instructions (Beyotime Biotechnology, C1062, Shanghai, China). To confirm our results, three independent experiments were conducted.

### Western blotting

Cells were lysed in a RIPA buffer (Beyotime Biotechnology), and total protein concentration was measured using a BIO-RAD Quick Start Bradford Dye Reagent (#500-0205; Bio-Rad Laboratories, Hercules, California, USA) according to the manufacturer’s instructions. Western blotting procedures were performed as reported previously [22]. Grayscale analysis was conducted using Image J software (National Institute of Health, Bethesda, Maryland, USA), and results were calculated from three independent experiments. The primary antibodies used in our experiments were as follows: YAP (ABclonal Biotech, A1002), Phospho-YAP (Cell Signaling Technology, #13008), LATS2 (Sigma-Aldrich, WH0007004M1), LATS1 (Bethyl laboratory, A300-477A; Montgomery, Texas, USA), MST1 (Cell Signaling Technology, #3682), 14-3-3β (Cell Signaling Technology, #9636), and GAPDH (Cell Signaling Technology, #5174).

### Statistical analysis

Results are presented as mean ± standard deviation. Comparisons between two groups were assessed using the unpaired Student’s *t* test. Cyclohexamide grayscale comparison was made using the paired *t* test. *P* values less than 0.05 were considered statistically significant. All statistical analyses were conducted using GraphPad Prism software (GraphPad Software, San Diego, California, USA).

## Results

### YAP regulated the proliferation and chemoresistance of osteosarcoma cells

**Fig. 1:**
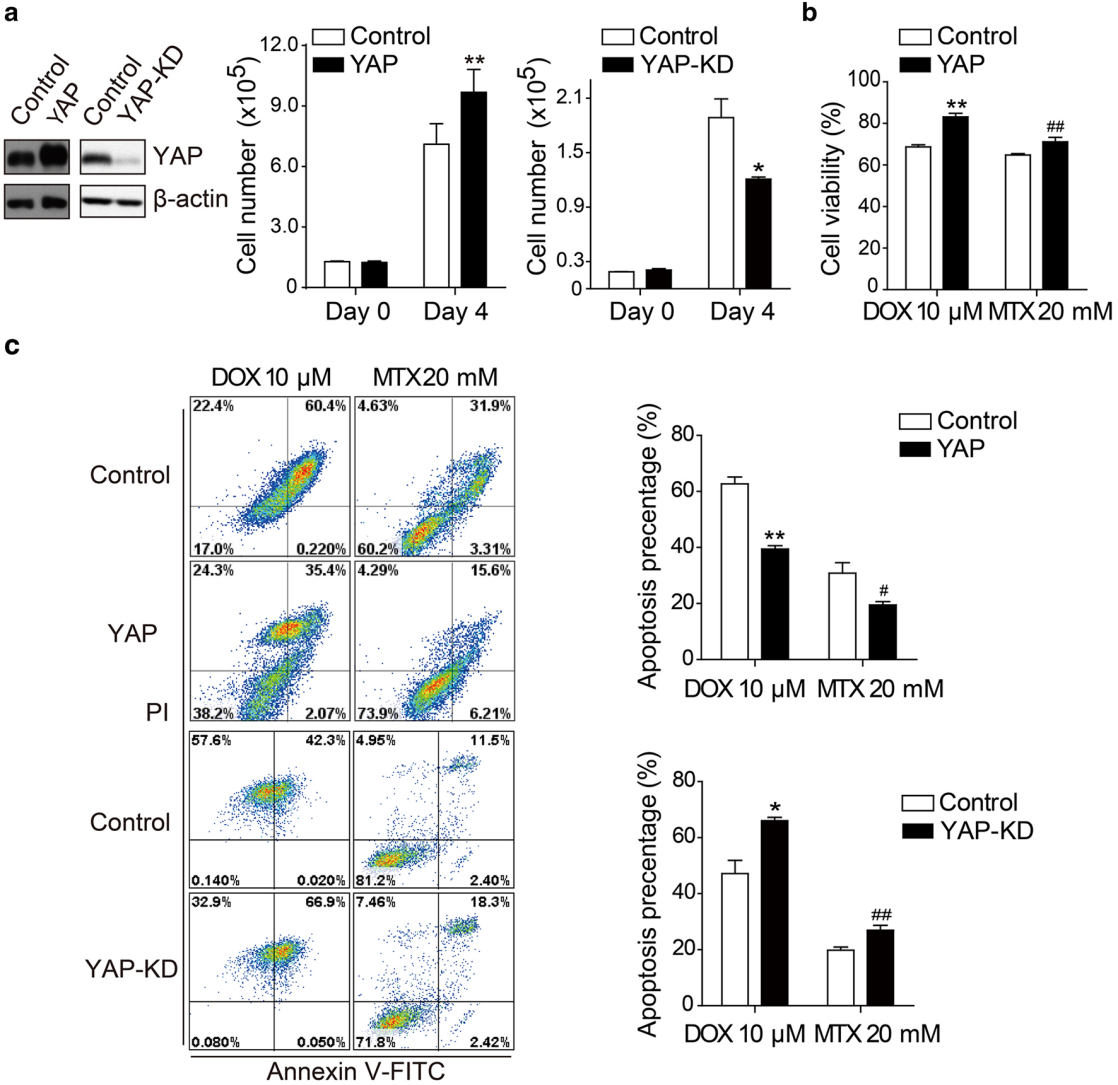
Yes-associated protein (YAP) increases the proliferation and chemoresistance of MG63 osteosarcoma cells. **a** Control and YAP-overexpressing/knockdown MG63 cells were seeded at the same concentration, and cell numbers were counted every 24 h. Data are shown as mean ± standard deviation (SD). Compared with control (*t* test, *n* = 3), **P < 0.01, and *P < 0.05. Results show that overexpression of YAP accelerated MG63 cell proliferation and knockdown of YAP decreased cell proliferation. **b** Cell viability was analyzed by detecting total cellular adenosine triphosphate in methotrexate (MTX) (20 mM)- or doxorubicin (DOX) (10 μM)-treated control and YAP-overexpressing MG63 cells. Data are shown as mean ± SD. Compared with control (*t* test, *n* = 3), **P < 0.01, and ^##^ *P* < 0.01. Overexpression of YAP increased the viability of MG63 cells treated with MTX (20 mM) or DOX (10 μM). **c** *Left panel* representative images of flow cytometry analysis of YAP-overexpressing and YAP-knockdown MG63 cells treated with MTX (20 mM) or DOX (10 μM) and stained by Annexin V-fluorescein isothiocyanate (FITC) and propidium iodide. Due to the natural fluorescence of DOX, we calculated only Annexin V-FITC-positive cells in DOX-treated cells. *Right panel* Quantitative analysis of apoptosis percentages according to the results of* left panel*. Data are shown as mean ± SD. Compared with control (*t* test, *n* = 3), *P < 0.05, **P < 0.01, ^#^ *P* < 0.05, and ^##^ *P* < 0.01. The flow cytometry results showed that YAP increased the chemoresistance of osteosarcoma cells and knockdown of YAP increased the chemosensitivity of osteosarcoma cells. YAP-KD, knockdown of YAP

To investigate the function of the Hippo/YAP pathway in osteosarcoma chemoresistance, we successfully established stable YAP-overexpressing and YAP-knockdown MG63 cell lines by retrovirus and lentiviral infection. As shown in Fig. 1a, overexpressing and knockdown of YAP resulted in accelerated and slowed cell proliferation, as detected by cell number counting. Moreover, cell viability assay showed that overexpression of YAP increased the viability of MG63 cells treated with high-concentration methotrexate (20 mM) or doxorubicin (10 μM) (Fig. 1b). Annexin V-FITC/PI staining and flow cytometry analysis confirmed the protective function of YAP in response to methotrexate (20 mM) or doxorubicin (10 μM), as the apoptosis of YAP-overexpressing cells was significantly lower than that of the control (*P* = 0.001 and *P* = 0.043, respectively). Additionally, YAP-knockdown cells demonstrated increased sensitivity to methotrexate and doxorubicin (Fig. 1c). Together, these data showed that YAP increased cell growth and the chemoresistance of osteosarcoma cells.

### Methotrexate and doxorubicin induced YAP activation in MG63 and U2OS osteosarcoma cells

**Fig. 2:**
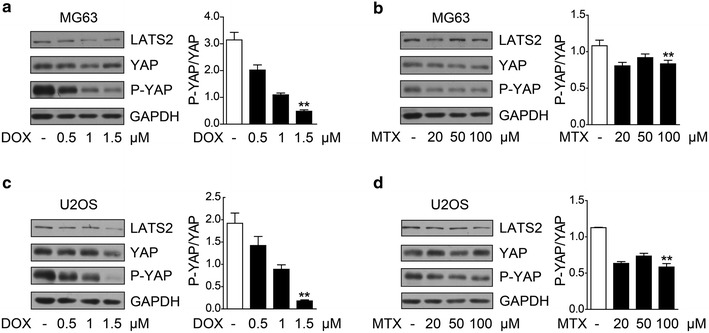
MTX and DOX induce YAP activation in MG63 and U2OS osteosarcoma cells. **a**, **b** *Left panel* Western blotting representative image of large tumor suppressor kinase 2 (LATS2), YAP, and YAP phosphorylation at Ser127 in MG63 cells with indicated concentrations of MTX or DOX treatments. *Right panel* Grayscale comparison of phosphorylated YAP to YAP total protein. Data are shown as mean ± SD. Compared with control (*t* test, *n* = 3), **P < 0.01. YAP phosphorylation level at Ser127 and LATS2 protein level in MG63 cells was decreased by MTX and DOX. **c**, **d** *Left panel* Western blotting representative image of LATS2, YAP, and YAP phosphorylation at Ser127 in U2OS cells with indicated concentrations of MTX or DOX treatments. *Right panel* Grayscale comparison of phosphorylated YAP to YAP total protein. Data are shown as mean ± SD. Compared with control (*t* test, *n* = 3), **P < 0.01. YAP phosphorylation level at Ser127 and LATS2 protein level in U2OS cells was decreased by MTX and DOX. *P-YAP* YAP phosphorylation

**Fig. 3:**
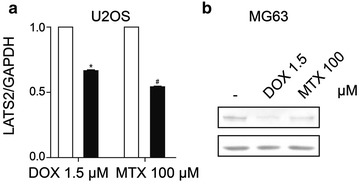
LATS1/2 total protein decreases in response to MTX/DOX treatment in osteosarcoma cells. **a** Grayscale comparisons of LATS2 total protein to that of GAPDH in U2OS cells, according to the western blotting results in Fig. 2c, d. The *error bars* represent mean ± SD. Compared with control (*t* test, *n* = 3), *P < 0.05 and ^#^ *P* < 0.05. LATS2 protein level was decreased by MTX and DOX in U2OS cells.** b** Representative image of LATS1 total protein in MG63 cells treated with indicated concentrations of MTX or DOX. LATS1 protein level was decreased by MTX and DOX in MG63 cells

To further investigate the role of the Hippo/YAP pathway in osteosarcoma chemoresistance, we evaluated LATS1/2 total protein level and Ser127 phosphorylation of YAP in osteosarcoma cells. Before Western blotting analysis, MG63 and U2OS were treated with methotrexate or doxorubicin at different concentrations for 24 h. We observed that LATS1/2 total protein decreased in osteosarcoma cells treated with methotrexate or doxorubicin (Figs. 2, 3). As shown in Fig. 2a and c, phosphorylation of YAP decreased significantly in a concentration-dependent manner after doxorubicin treatment. Similar results were found after treatment of methotrexate (Fig. 2b, d). Grayscale comparison of western blotting results showed that both methotrexate and doxorubicin could induce YAP activation, suggesting that YAP plays a role in osteosarcoma chemoresistance.

### Methotrexate and doxorubicin induced YAP nuclear translocation

**Fig. 4:**
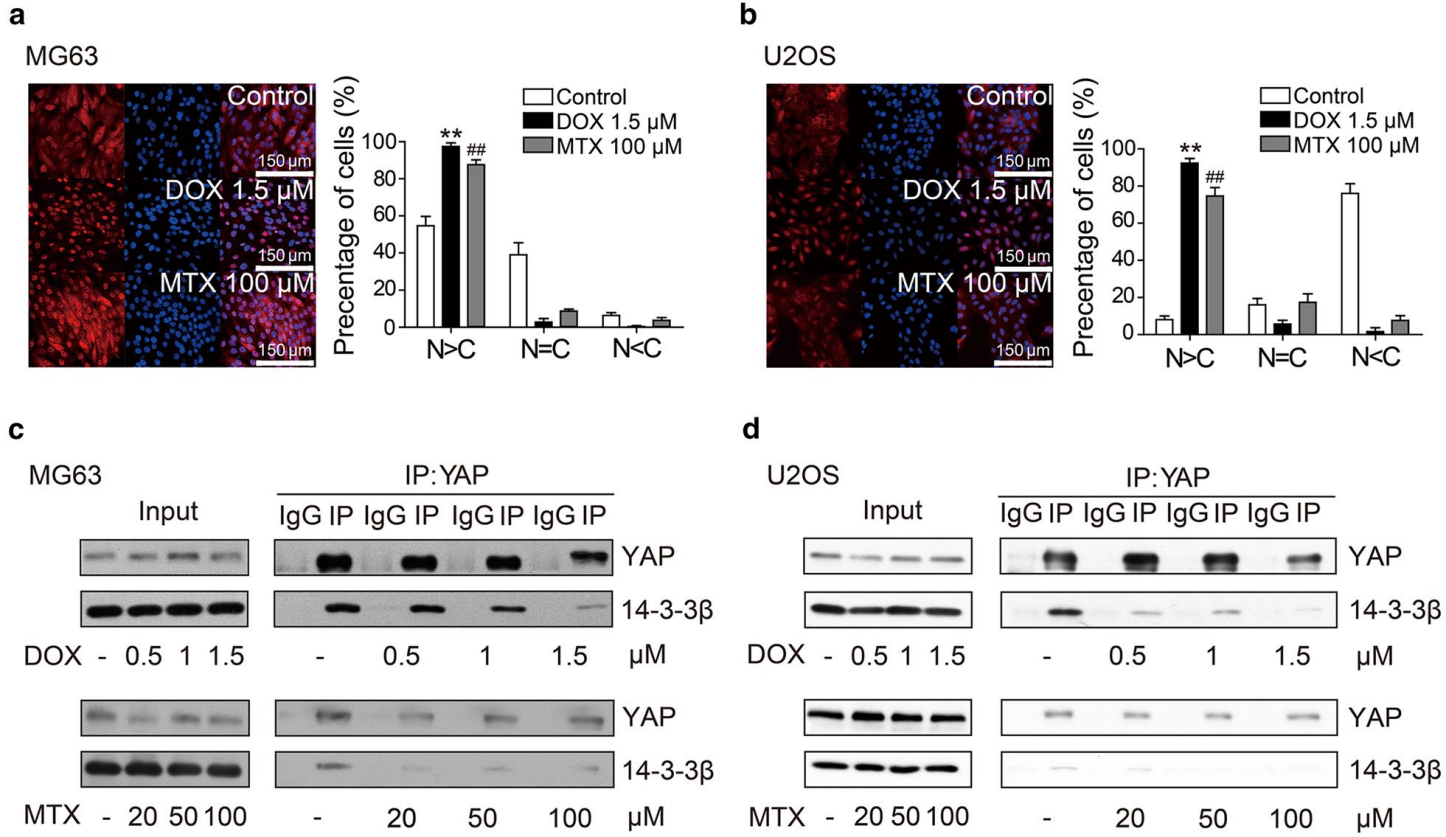
MTX and DOX induce YAP nucleus translocation. **a**, **b** MG63 and U2OS cells cultured on coverslips were exposed to MTX (100 μM) or DOX (1.5 μM) for 24 h. Endogenous YAP was stained using an anti-YAP antibody (*red*), and nuclei were stained with DAPI (*blue*). The subcellular localization of YAP was quantified (*lower panels*). The *error bars* represent mean ± SD. Compared with control (*N* nucleus; *C* cytoplasm. *t* test, *n* = 100), **P < 0.01, ^##^ *P* < 0.01. Results show that MTX and DOX promoted YAP nuclear translocation. **c**, **d** Co-immunoprecipitation was applied to investigate the interaction between YAP and 14-3-3β in MG63 and U2OS cells. Cells were exposed to indicate concentrations of MTX or DOX for 24 h before co-immunoprecipitation. The results were determined by western blotting and the interaction between YAP and 14-3-3β was decreased by MTX and DOX

Since YAP phosphorylation at Ser127 determines its location in either the cytoplasm or the nucleus [23], using immunofluorescence staining we examined the intracellular location of YAP in methotrexate- or doxorubicin-treated MG63 and U2OS cells. We found that YAP translocated to the nucleus in both MG63 and U2OS cells after methotrexate or doxorubicin treatment (Fig. 4a, b). 14-3-3 protein is well known for its critical role in inhibiting the nuclear translocation of YAP. To further validate the effect of methotrexate and doxorubicin on YAP activity, we determined the interaction between YAP and 14-3-3. Cells exposed to methotrexate or doxorubicin were harvested for co-immunoprecipitation studies. Consistent with the results from the immunofluorescence staining assays, methotrexate and doxorubicin dramatically reduced the interaction between YAP and 14-3-3 (Fig. 4c, d). Methotrexate and doxorubicin decreased the interaction between YAP and 14-3-3β and induced YAP nuclear translocation, indicating that both are capable of activating YAP.

### Methotrexate and doxorubicin decreased MST1 expression by altering its protein stability

**Fig. 5:**
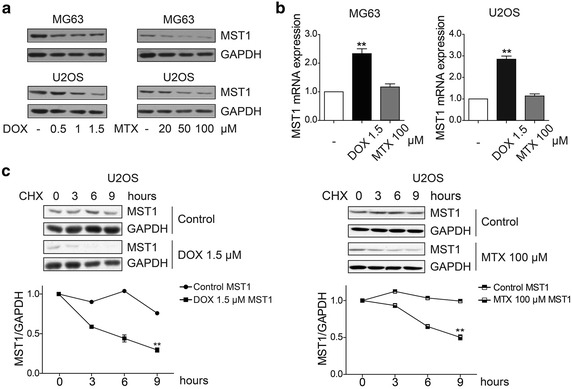
MTX and DOX decrease mammalian sterile 20-like kinase 1 (MST1) expression by altering its protein stability. **a** Western blotting representative image of mammalian sterile 20-like kinase 1 (MST1) total protein level in MTX- or DOX-treated osteosarcoma cells. MST1 protein level was decreased by MTX and DOX. **b** The MST1 mRNA levels in the control and MTX- or DOX-treated osteosarcoma cells were monitored via real-time polymerase chain reaction using specific primers. The *error bars* represent mean ± SD. Compared with control (*t* test, *n* = 3), **P < 0.01. MST1 mRNA did not decrease after MTX or DOX treatments. **c** U2OS cells were treated with 100 μg/mL of cycloheximide for the indicated periods. The endogenous MST1 protein levels were determined by western blotting (*upper panels*), and relative MST1 protein levels were normalized to those of GAPDH (*lower panels*). The *error bars* represent mean ± SD. Compared with control (paired *t* test, *n* = 3), **P < 0.01.MST1 degradation was increased by MTX and DOX in U2OS cells. *CHX* cycloheximide

MST1 is a key component of the Hippo signaling pathway. To better understand MST1’s role in osteosarcoma chemoresistance, we examined its protein level. As shown in Fig. 5a, total protein of MST1 remarkably declined in methotrexate- or doxorubicin-treated osteosarcoma cells. However, after methotrexate and doxorubicin treatment, we did not observe the down-regulation of MST1 mRNA level (Fig. 5b), suggesting that doxorubicin and methotrexate reduce MST1 expression at the protein level. Then, U2OS cells were treated with cycloheximide (a common inhibitor of protein biosynthesis in eukaryotic organisms) for the times indicated and harvested for analysis of the MST1 protein level. As shown in Fig. 5c, methotrexate and doxorubicin accelerated degradation of MST1, suggesting that MST1 is destabilized by methotrexate and doxorubicin, which is responsible for the activation of YAP induced by methotrexate and doxorubicin.

## Discussion

**Fig. 6:**
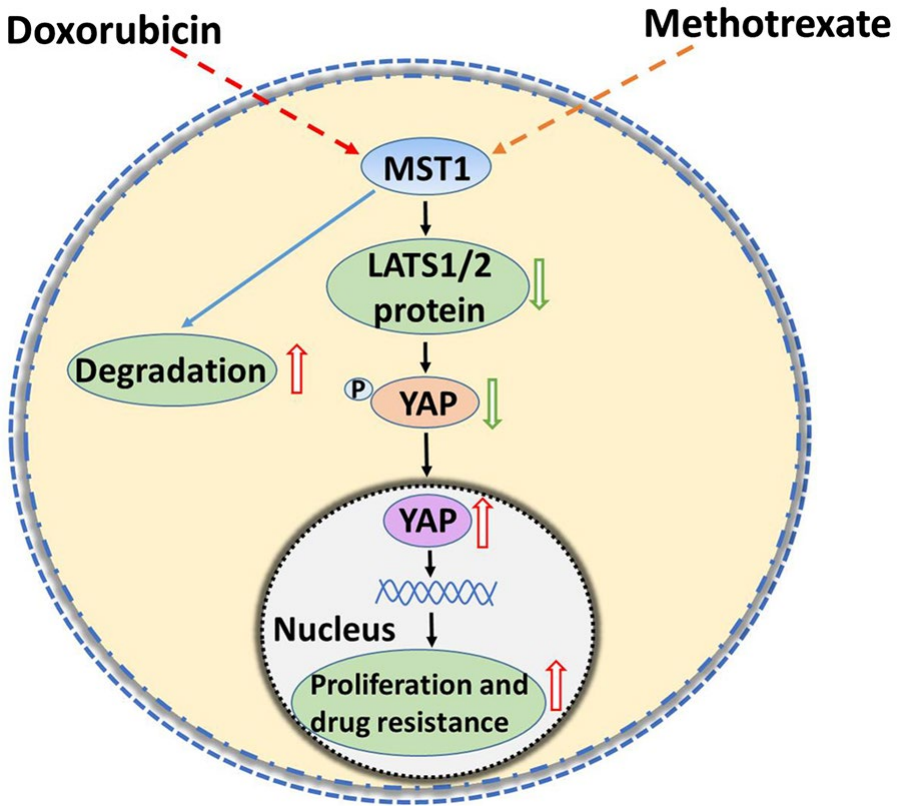
Systematic model of the Hippo/YAP signaling pathway affecting cytotoxic drug resistance in osteosarcoma cells. Methotrexate and doxorubicin increase MST1 degradation in osteosarcoma cells, decreasing LATS1/2 total protein level and YAP phosphorylation, resulting in enhanced nuclear translocation of YAP. Endonuclear YAP then promotes the transcription of its downstream targets involved in anti-apoptosis and proliferation, leading to elevated proliferation and resistance to methotrexate and doxorubicin

Although many reports have shown that the Hippo/YAP signaling pathway is involved in tumorigenesis, little is known about its role in osteosarcoma chemoresistance. In the present study, we showed that, in osteosarcoma cells, methotrexate and doxorubicin activated YAP, promoting its nuclear translocation by accelerating MST1 protein degradation and decreasing LATS1/2 protein level. Furthermore, YAP regulated the proliferation and chemoresistance in MG63 osteosarcoma cells, indicating that the Hippo/YAP pathway plays a role in osteosarcoma chemoresistance (Fig. 6).

Current management of osteosarcoma patients focuses on neoadjuvant chemotherapy plus surgery. However, many patients die from tumor metastases because of poor response to chemotherapy. In the past 10 years, several cell signaling pathways, including phosphoinositide 3 kinase (PI3 K)/Akt, extracellular signal-regulated kinase (ERK)1/2, Notch, and Wnt-β-catenin, have been identified to be involved in osteosarcoma chemoresistance [24–26]. Recently, the Hippo/YAP signaling pathway has been shown to modulate organ size [9, 27]. Moreover, other studies have shown that YAP promotes neoplastic cell proliferation and accelerates oncogenic senescence [28, 29]. Mao et al. [19] showed that SIRT1 increases the interaction between YAP2 and TEAD4 and enhances resistance to the anti-cancer drug cisplatin by deacetylating YAP2 in HCC cells. Phosphorylation-defective YAP overexpression makes ovarian cancer cells much more resistant to cisplatin [30]. Nevertheless, the relationship between osteosarcoma chemoresistance and the Hippo/YAP signaling pathway is still unclear. To our knowledge, our study is the first to focus on the function of the Hippo/YAP signaling pathway in osteosarcoma chemoresistance. We found that, with methotrexate and doxorubicin treatment, YAP increases MG63 cell proliferation and cytotoxic survivability. Accordingly, methotrexate and doxorubicin inhibit the phosphorylation of YAP. Similar to doxorubicin, cisplatin also inhibits YAP phosphorylation at Ser 127 in HCC cells [19]. Activated YAP then translocates to the nucleus and enhances the chemoresistance of osteosarcoma cells.

We also determined that reduced MST1 and LATS1/2 protein level in response to methotrexate and doxorubicin may cause up-regulation of YAP activity. Previously, our colleagues [31] reported that c-Abl stabilizes MST1 protein level and protects it from ubiquitination by phosphorylating MST1 at Y433 in HEK 293T and Neuro2A cells. In addition, Ren et al. [32] found that proteasome-mediated down-regulation of MST1 by heat shock protein 70 enhances resistance to cisplatin in prostate cancer cells. Autophagy is another regulated pathway of cellular degradation. In various tumor cells, increased autophagy has shown protective effects against cytotoxic agents [33, 34]. MST1/2 directly phosphorylates LC3 and enhances the cell autophagy process [35]. Therefore, decrease of MST1 could decreases cell autophagy and then improve osteosarcoma chemosensitivity. As there is no evidence for lysosome-mediated degradation of MST1, we could not confirm whether proteasomal or lysosomal degradation is responsible for the decrease of MST1 protein level in osteosarcoma cells treated with chemotherapeutic drugs. Further experiments are needed to address this.

## Conclusions

In conclusion, our results suggest that the Hippo/YAP signaling pathway induces osteosarcoma chemoresistance. The reduction in the concentration of MST1 and LATS1/2 proteins by methotrexate and doxorubicin leads to YAP activation and nuclear translocation. Moreover, YAP increases the proliferation and methotrexate/doxorubicin resistance in MG63 cells. Taken together, our findings suggest that the decrease of YAP may improve osteosarcoma chemosensitivity.
